# Implementation of +PERTO^®^ in Rehabilitation for Total Knee Arthroplasty: A Pilot Study

**DOI:** 10.3390/healthcare13060605

**Published:** 2025-03-11

**Authors:** Tiago Emanuel Soares de Araújo, Elsa Paula Santos Rodrigues, Ana Raquel Varejão Files, João Miguel Almeida Ventura-Silva, Jorge Eduardo Ferreira Mendes, André Filipe Morais Pinto Novo, Olga Maria Pimenta Lopes Ribeiro

**Affiliations:** 1 Abel Salazar Institute of Biomedical Sciences, University of Porto, 4050-313 Porto, Portugal; 2 Tâmega e Sousa Local Health Unit, 4560-136 Penafiel, Portugal; elsa.justdoit@gmail.com (E.P.S.R.); ana_files@hotmail.com (A.R.V.F.); 70783@chts.min-saude.pt (J.E.F.M.); 3 RISE@Health, 4200-319 Porto, Portugal; joao.ventura@essnortecvp.pt (J.M.A.V.-S.); olgaribeiro@esenf.pt (O.M.P.L.R.); 4 Northern Health School of the Portuguese Red Cross, 3720-126 Oliveira de Azeméis, Portugal; 5 Research Center for Active Living and Wellbeing–LiveWell, 5300-253 Bragança, Portugal; andre@ipb.pt; 6 School of Health, Polytechnic Institute of Bragança, 5300-253 Bragança, Portugal; 7 Porto Nursing School, 4200-072 Porto, Portugal

**Keywords:** total knee arthroplasty, rehabilitation nursing, eHealth, digital interventions

## Abstract

Background/Objectives: Total knee Arthroplasty (TKA) is a prevalent treatment modality for degenerative knee diseases. Nevertheless, the success of the intervention is contingent on effective rehabilitation. The +PERTO^®^ program (a Technological Rehabilitation Nursing Program) was developed as a mobile application comprising three phases to support patients during the perioperative period by providing exercises, information, and direct communication with healthcare professionals. The present study aims to evaluate the effects and usability of the +PERTO^®^ program in patients undergoing total knee arthroplasty. Methods: In 2024, a hospital in northern Portugal conducted a pilot trial with eleven patients undergoing elective total knee arthroplasty. Researchers collected data both before surgery and six weeks after discharge. To evaluate effectiveness, software satisfaction, and usability, they used several assessment tools, including the Oxford Knee Score (OKS), SF-36v2, Visual Analog Scale (VAS), Short Physical Performance Battery (SPPB), QSEnf-10, and System Usability Scale (SUS). Researchers analyzed the data using both descriptive and inferential statistics. The hospital’s ethics committee and board of directors approved the study. Results: There was a significant reduction in pain (*p* = 0.041) and improvement in knee functionality (*p* = 0.010), physical performance (*p* = 0.038), and quality of life (*p* < 0.05). Patient satisfaction was high (QSEnf-10: 3.8/4), and the usability of +PERTO^®^ was considered excellent (SUS: 96.6/100). Conclusions: The +PERTO^®^ program proved to be an effective and innovative solution to support rehabilitation after TKA, promoting improvements in pain, functionality, and quality of life. This digital program stands out for its high rate of usability and its ability to modernize healthcare by providing a patient-centered approach.

## 1. Introduction

Over the past decade, with population aging and increased longevity, the growing prevalence of degenerative diseases has significantly affected self-care and, consequently, the quality of life of millions of people worldwide [1,2]. Osteoarthritis is a progressive condition that wears down cartilage in the joints, leading to chronic pain, stiffness, and limited movement, especially in weight-bearing joints like the knee [3].

Age, obesity, history of trauma, and genetic predisposition are aspects that contribute to the progression of these diseases. When conservative treatments fail to alleviate symptoms or restore function, total knee arthroplasty is an effective intervention, replacing the damaged joint with a prosthetic implant. This surgical approach not only relieves pain but also improves the mobility and functionality of the knee. The rising number of total knee arthroplasty procedures highlights the necessity of creative methods for postoperative rehabilitation and training [4]. Comprehensive rehabilitation is necessary for functional recovery and patient adaptation to their new state. Surgical intervention alone does not guarantee a complete restoration of function or quality of life [5].

Alongside traditional rehabilitation methods, recent studies show that innovative approaches can improve outcomes for total knee arthroplasty patients. With advancements in digital technology, personalized rehabilitation strategies, and new treatment methods, recovery is being enhanced [6].

It is important to highlight that traditionally, rehabilitation for individuals undergoing total knee arthroplasty has been carried out with the physical presence of a rehabilitation professional. However, to facilitate patients’ prompt return home, developing new care models that accommodate shorter hospital stays is essential. There has been growing interest in eHealth technologies and mobile applications to support care, especially rehabilitation, in response to this need and technological advancements, particularly those accelerated by the COVID-19 pandemic. This allows for remote access and continuity of care [7,8,9,10].

In this context, eHealth tools are as effective as traditional care for pre- and post-operative rehabilitation in total knee arthroplasty patients, providing significant physical, psychological, and social benefits [11]. The use of these technological devices has been crucial in educating patients on recovery, guiding regular and appropriate exercise, and monitoring pain progression, activity levels, and exercise adherence. It is also worth noting the one-way or two-way communication with health professionals to facilitate continuity of care and goal setting [12].

In this context, the +PERTO^®^ program is a pioneering initiative in the Portuguese healthcare system, offering comprehensive perioperative patient monitoring via a mobile application. While some digital interventions support postoperative recovery after total knee arthroplasty [13,14,15], the +PERTO^®^ program is unique because it provides monitoring throughout all three perioperative phases: Phase 1 (preoperative, before hospitalization), Phase 2 (hospitalization), and Phase 3 (postoperative, after returning home) [16].

In Phase 1, the preoperative stage, which begins 15 to 30 days before surgery, patients attend a consultation with their caregiver or family member. During this visit, they gain access to the +PERTO^®^ program. They then follow a daily exercise program, receive important information in stages, and communicate directly with the healthcare team. This phase focuses on self-care, surgical preparation, recovery, and rehabilitation.

In Phase 2, during hospitalization, the healthcare facility offers patients a personalized rehabilitation protocol and detailed instructions for its implementation. The +PERTO^®^ program helps with recovery in Phase 3, after patients have been discharged, by offering daily exercise routines, motivational messages, and tools to manage common postoperative issues like pain, swelling, and wound care [16]. The capabilities of the +PERTO^®^ program ensure a remotely monitored and safe recovery during this phase.

In Portugal, while the needs of total knee arthroplasty patients before surgery and after discharge are well understood, access to proper care remains limited. Barriers such as limited healthcare accessibility and geographic distance often prevent patients from receiving necessary support. As a result, complications and delays in functional recovery are common, frequently leading to emergency care visits.

To address these challenges, researchers developed the +PERTO^®^ program to provide continuous, personalized postoperative care [16]. By integrating physical, psychological, and surgical support, the +PERTO^®^ program aims to create a more connected and patient-centered healthcare experience. Its innovative approach aims to improve surgical outcomes and enhance the quality of life for patients and caregivers during the perioperative period.

In line with the World Health Organization’s (WHO’s) recommendations on digital interventions for strengthening the healthcare system, it is essential, from the early stages of implementation, to assess their usability and effectiveness, aiming to identify opportunities for improvement, both in terms of technological performance and patient behavioral performance or engagement [17].

In line with the aforementioned, this study aims to evaluate the effects and usability of the +PERTO^®^ program, a Technological Rehabilitation Nursing Program, in patients undergoing total knee arthroplasty.

## 2. Materials and Methods

### 2.1. Study Design

This study used a pre-experimental pilot design, including pre- and post-assessments, and was conducted with a single cohort of patients undergoing total knee arthroplasty at a hospital in northern Portugal. The study spanned a four-week preoperative phase and a six-week post-discharge phase, running from January to March 2024.

### 2.2. Participant Selection

Researchers used a non-probability convenience sampling method to select participants, as this was their first experience implementing the +PERTO^®^ program. They invited patients scheduled for elective, primary, and unilateral total knee arthroplasty to participate.

To participate in the study, patients needed to meet the following criteria: (1) have a scheduled primary total knee arthroplasty, (2) have no history of consciousness or cognitive impairments, based on the Glasgow Coma Scale and Mini-Mental State Examination, (3) be able to read and write, (4) own and use a smartphone or internet-enabled device, and (5) have a designated caregiver or family member for support. Researchers excluded patients who had undergone total hip replacement or had conditions that prevented physical activity.

Given the exploratory nature of this pilot study, researchers recruited 11 participants in December 2023, all of whom had total knee arthroplasty scheduled between January and March 2024.

### 2.3. Intervention

**Figure 1:**
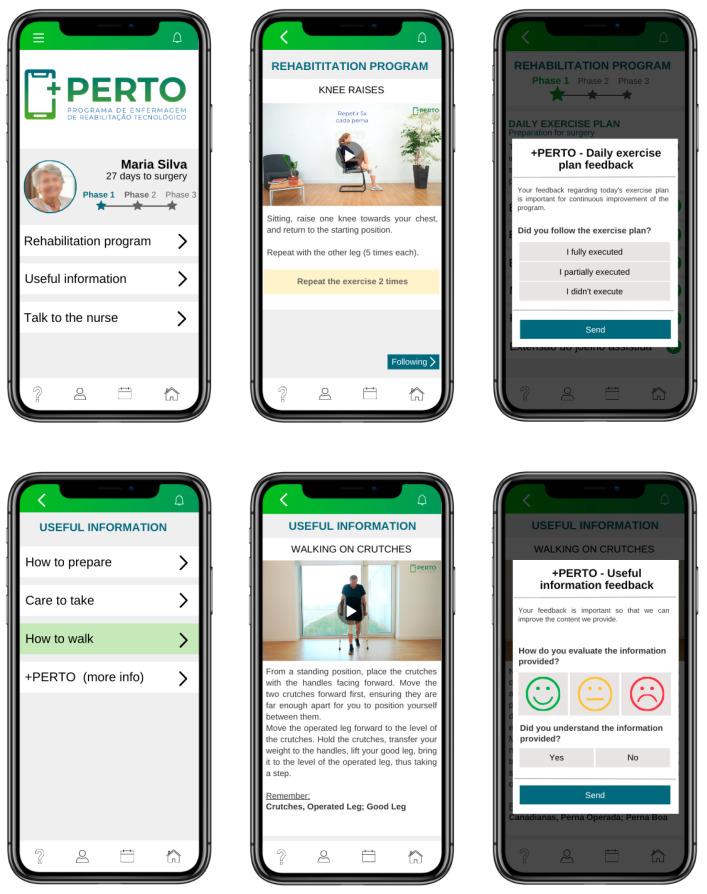
The +PERTO^®^ Program: a Technological Rehabilitation Nursing Program.

Researchers implemented the +PERTO^®^ program, a Technological Rehabilitation Nursing Program, developed with the help of digital technology, as part of the intervention. Rehabilitation experts have validated the content of the program’s three phases (Figure 1) [11].

The study participants used the +PERTO^®^ program throughout the three phases of the perioperative period, as detailed in Table 1. During the preoperative appointment, healthcare providers instructed the patients on how to use the application, and they began the daily physical exercise program. The application progressively provided useful information and facilitated direct communication with the healthcare team (Phase 1). The app delivered relevant information about the anatomical composition of the knee, the surgical procedure and anesthetic regimen, prescribed medications, and strategies for self-care impacted by the surgery, along with guidance for discharge preparation. After surgery, during hospitalization (Phase 2), the app provided patients with ongoing access to information as they followed the institution’s recommended in-person rehabilitation protocol.

In the postoperative period (Phase 3), which extends from the day of discharge until six weeks later, patients utilize the +PERTO^®^ program’s mobile application to facilitate ongoing rehabilitation exercises at home. This application offers daily exercise regimens, motivational messages, and decision-support algorithms focused on pain management, edema, and surgical wound care. The mobile application closely monitors adherence to the program by providing automated feedback (Table 1).

### 2.4. Study Variables and Data Collection Instruments

The present study examined the relationship between patient-reported pain and various sociodemographic and clinical variables. The study population included patients undergoing total knee arthroplasty. Researchers studied the following variables: age, gender, education, marital status, profession, employment status, pain, knee functionality, and physical performance. Additionally, they assessed quality of life, satisfaction with rehabilitation nursing care, and usability. The study used the Visual Analogue Scale (VAS) to monitor the intensity of pain perceived by patients. The VAS is a 10-cm line with the endpoints marked by “no pain” and “maximum imaginable pain”. Researchers selected VAS for its ability to detect variations in pain perception over time. They employed the Oxford Knee Score (OKS) in its Portuguese version to evaluate knee function and pain in patients undergoing TKA. This instrument comprises 12 items, with scores ranging from 0 to 4, resulting in a total score ranging from 0 to 48, where higher scores indicate better function and less pain. Researchers selected the OKS due to its sensitivity to postoperative changes [18]. To ensure data reliability, researchers used the scoring method recommended by Oxford University Innovation Limited [19].

The Short Physical Performance Battery (SPPB) was used to assess physical performance through tests of balance, walking speed, and leg strength (getting up and sitting down from a chair). The score ranges from 0 to 12, with higher scores indicating better physical performance. Each test is scored from 0 to 4, resulting in a maximum score of 12, which corresponds to good physical performance [20]. Researchers selected this instrument for its relevance in assessing physical functionality and for its ability to predict health outcomes.

Researchers assessed health-related quality of life using the SF-36 Health Survey (SF36v2). They translated and validated it for the Portuguese population. This instrument encompasses the following eight domains, namely: physical functioning, physical role, bodily pain, general health, vitality, social functioning, limitations due to emotional problems, and mental health. The scores for each domain range from 0 to 100, with higher scores indicating better health [21]. Researchers chose the v2 version due to its wide use and ability to capture varied aspects of patients’ health.

The Rehabilitation Nursing Care Satisfaction Questionnaire (QSEnf-10) assesses patient satisfaction with the rehabilitation nursing care they receive through its 10-item instrument. Each item is answered on a Likert scale ranging from 1 to 4 (1 = very dissatisfied, 4 = very satisfied), with higher total scores indicating greater satisfaction. The researchers selected this instrument for its specificity and relevance in assessing patients’ perceptions of rehabilitation care [22].

The System Usability Scale (SUS) is a widely used instrument for assessing the usability of digital systems and applications. Developed in 1986, it is characterized by its simplicity, ease of use, and cross-cultural adaptability, having been translated and validated into European Portuguese [23,24]. Researchers selected it for its recognized validity in measuring the usability of digital applications. The scale consists of 10 items, and participants respond using a 5-point Likert scale, where 1 means “strongly disagree” and 5 means “strongly agree”. The total score ranges from 0 to 100, with higher scores indicating better usability. The instrument was segmented into two sub-scales/dimensions: usability (items 1, 2, 3, 5, 6, 7, 8, and 9) and learnability (items 4 and 10) [24].

To calculate the final score, researchers sum the scores for each item, contributing on a scale of 1 to 5. For items 1, 3, 5, 7, and 9, they determined the individual score by subtracting 1 from the mark received. For items 2, 4, 6, 8, and 10, they calculated the contribution by subtracting the mark received from 5. To obtain the final SUS score, researchers multiplied the total scores by 2.5. After computing the final SUS score, researchers converted it into percentiles (0–100) and assigned letter grades from F to A+. A grade of A indicates “excellent performance”, C represents “average performance”, and F signifies “poor performance” [25].

Researchers ensured comparability between the usability and learnability dimensions and the overall SUS score (0 to 100) by multiplying the total contributions of each dimension by 3.125 and 12.5, respectively. As a result, the adjusted usability dimension score ranges from 0 to 100 in increments of 3.125, while the learnability dimension score ranges from 0 to 100 in increments of 12.5 [26].

### 2.5. Data Collection Procedure

Researchers assessed participants at two distinct time points: at the start of the study (T0) and six weeks after hospital discharge (T1). They evaluated the digital intervention by comparing data collected at the beginning of the study (T0) with data obtained after the intervention (T1). This methodological approach analyzed participants’ progress over time. It offered a thorough evaluation of how effective and relevant the +PERTO^®^ program is in preparing patients for total knee arthroplasty and helping them recover after surgery.

During the initial assessment (T0), conducted between four and two weeks before surgery, researchers obtained informed consent, collected sociodemographic data and personal history, and characterized the caregiver or family caregiver. The sociodemographic variables included age, gender, education level, marital status, occupation, and employment status. Researchers assessed clinical variables such as pain, knee functionality, and physical performance using the Visual Analogue Scale (VAS), Oxford Knee Score (OKS), and Short Physical Performance Battery (SPPB), respectively. To evaluate the quality of life, researchers used the SF-36 Health Survey, version 2 (SF36v2).

In the final evaluation (T1), six weeks after discharge, researchers repeated the clinical assessments and measured satisfaction and usability. Researchers used the Rehabilitation Nursing Care Satisfaction Questionnaire (QSEnf-10) and the System Usability Scale (SUS) to assess satisfaction and usability. All participants completed the evaluations at both times, ensuring the integrity of the data obtained.

### 2.6. Data Analysis Procedure

Researchers analyzed the data using descriptive and inferential statistics with SPSS software (version 29.0). Quantitative variables were described using the minimum, maximum, mean, standard deviation (±), median, and interquartile ranges. Categorical variables were summarized with absolute and relative frequencies.

Before conducting the inferential analysis, researchers assessed the normality of the distributions using the Shapiro–Wilk test. Given the limited sample size and non-normal data distribution, they applied non-parametric statistical tests. To compare assessment times (T0 and T1) and evaluate the intervention’s effect, researchers used the Wilcoxon test for paired samples. They set the significance level at 5% (*p* < 0.05). The Wilcoxon test was chosen because it does not require normal assumptions and is more robust for small, asymmetrically distributed samples. Despite the limited sample size, this approach enhanced the internal validity of the analysis.

### 2.7. Ethical Considerations

The Ethics Committee of the hospital approved the study, and it was carried out under reference number 27/2023, dated 28 April 2023. After researchers explained the study and its objectives, all participants signed an informed consent form.

## 3. Results

The present study involved 11 patients who underwent total knee arthroplasty, with a mean age of 65.55 years (SD = 4.97). Most of the participants were female (*n* = 9; 81.8%), married (*n* = 9; 81.8%), and retired (*n* = 9; 81.8%). Most participants resided with another person (*n* = 10, 90.9%), predominantly with their spouse (*n* = 7, 63.6%). The median number of children was two, with a maximum of three and a minimum of two.

Although the implementation of the +PERTO^®^ program began 30 to 15 days before the surgical procedure and lasted up to 6 weeks postoperatively, the adherence rate in all three phases was 100%.

The study assessed clinical variables (pain, knee functionality, and physical function) and quality of life at baseline (T0) and six weeks after hospital discharge (T1). Concerning the experience of pain, the mean score on the Visual Analogue Scale (VAS) exhibited a decrease from 3.5 (SD = 2.1) at T0 to 1.2 (SD = 1.7) at T1. The Wilcoxon test showed a significant decline in pain intensity (Z = −2.044; *p* = 0.041; r = 0.62) after the +PERTO^®^ program’s intervention.

Regarding knee functionality, the results of the Wilcoxon test indicated significant differences in two of the three domains assessed by the Oxford Knee Score (OKS) between the preoperative consultation (T0) and the follow-up consultation after six weeks (T1) (Table 2). The “pain” domain of the OKS exhibited a statistically significant difference (Z = −2.807; *p* = 0.005; r = 0.85), signifying a substantial reduction in knee pain experienced by patients following treatment/the +PERTO^®^ program. A similar trend was observed in the “OKS Final Score”, which also exhibited a significant difference (Z = −2.585; *p* = 0.010; r = 0.78), suggesting an enhancement in knee function and quality of life for the patients. In contrast, the “function” domain showed no significant difference (Z = −1.387; *p* = 0.165; r = 0.42), suggesting that physical function improved more slowly or inconsistently than pain and the overall assessment.

The Short Physical Performance Battery (SPPB) results (Table 2) demonstrated a significant improvement in the Total Balance Test between the preoperative period (T0) and six weeks post-intervention (T1) (Z = −2.070, *p* = 0.038; r = 0.62), indicating a positive trajectory. However, no statistically significant differences were observed in the chair stand test (Z = −0.844, *p* = 0.399; r = 0.25), gait speed test (Z = −0.061, *p* = 0.951; r = 0.02), or total SPPB score (Z = −1.592, *p* = 0.111; r = 0.48). Although, the median total score increased from 7.00 to 9.00 and the chair stand test showed some improvement, suggesting that enhancements in other aspects of physical performance may require a longer duration or a more intensive intervention to achieve statistical significance.

The SF36v2 analysis (Table 2) highlights changes in health-related quality of life between the preoperative visit (T0) and six weeks post-discharge (T1). The Wilcoxon test was utilized to compare the scores across the various domains of the SF36v2, thereby identifying significant improvements following the implementation of the +PERTO^®^ program. Table 2 presents the median values and interquartile ranges (IQR) for each domain at the two assessment times, along with the Z and *p* values associated with each comparison. These values indicate the dimensions with significant differences.

The results of the SF-36v2 assessment indicate that the +PERTO^®^ program significantly improved patients’ quality of life following total knee arthroplasty, particularly in the domains of physical functioning, role limitations due to physical health, bodily pain, general health, vitality, and changes in health status. In contrast, the domains of social functioning, emotional role limitations, and mental health did not exhibit significant improvements, suggesting that alternative or complementary interventions may be necessary to optimize patient outcomes.

In relation to satisfaction with rehabilitation nursing care, the mean score on the QSEnf-10 scale was 3.8 (SD = 0.4) (see Table 3). This finding indicates a high level of satisfaction, with most patients categorizing themselves as being between “satisfied” and “very satisfied”. This lends further support to the hypothesis that the +PERTO^®^ rehabilitation program is relevant in meeting patients’ needs and promoting a positive experience during postoperative recovery.

At the initial stage (T1), the usability of the system was evaluated using the System Usability Scale (SUS). The mean score obtained, after conversion using Brooke’s formula, was 96.6 (SD = 3.4), with a range from 90 to 100 (see Table 4). This categorizes the +PERTO^®^ program as having “excellent performance” (grade A).

Regarding the dimensions of usability and learnability (see Table 5), the +PERTO^®^ program obtained mean scores of 96.3 (SD = 3.37) and 97.7 (SD = 7.5), respectively.

The results confirm that the +PERTO^®^ program is a technologically advanced tool that is easy to learn and has consistently received high ratings for usability and acceptance in the evaluated fields.

## 4. Discussion

Resulting from the initial experience of implementing the +PERTO^®^ program, although in Phase 3 the follow-up period for patients undergoing total knee arthroplasty was limited to six weeks, this pilot study, in addition to assessing the effects and usability, served the purpose of identifying the need for improvements of the +PERTO^®^ program.

Thus, in line with the defined objective, the results of this study showed that the +PERTO^®^ program had a positive impact on the postoperative recovery of patients undergoing total knee arthroplasty, with significant improvements in pain, functionality, and quality of life.

The findings concerning pain, as measured by the VAS and the pain domain of the OKS, demonstrate that the +PERTO^®^ program significantly contributes to the alleviation of postoperative pain. This finding aligns with the conclusions of previous studies, which underscored the significance of structured and continuous rehabilitation in the recovery process following total knee arthroplasty. Mobile applications have shown significant potential in reducing postoperative pain in patients undergoing total knee arthroplasty by offering tools for pain monitoring and management [27].

The combined effects on function, as evidenced by improvements in the functional domain of the Oxford Knee Score and the “total balance” domain of the Short Physical Performance Battery (SPPB), suggest that the +PERTO^®^ program not only alleviated pain but also facilitated progressive functional recovery. This positively influenced patients’ ability to return to daily activities and enhanced their quality of life. In a previous study, the authors demonstrated that the “RECOVER-E” application effectively improved physical function in patients undergoing total knee arthroplasty. The application supported rehabilitation by offering personalized exercises and incentives to motivate patients throughout their recovery [27].

The findings from the SF-36v2 survey indicate that the +PERTO^®^ program significantly impacts the quality of life for patients who have undergone total knee arthroplasty. The improvements are particularly pronounced in the domains of physical functioning, role limitations due to physical health, bodily pain, general health, vitality, and changes in health status, highlighting the program’s effectiveness in addressing functional and physical aspects directly related to postoperative recovery. A study in China found that using a mobile application after surgery could effectively improve knee function, encourage early adherence to functional exercises, and enhance the quality of life for patients undergoing total knee arthroplasty [28].

In contrast, no statistically significant differences were observed in the domains of social functioning, emotional role limitations, and mental health. This finding suggests that, although the program had a positive impact on physical aspects, targeted interventions addressing the emotional and social components of rehabilitation may be necessary to achieve a more comprehensive recovery. The incorporation of additional strategies, such as psychological support or social-interaction-enhancing activities, may be considered to improve outcomes in these domains.

The present study corroborates the evidence presented by Wang et al. (2023), who demonstrated that mobile app-based rehabilitation programs have the potential to improve patients’ self-efficacy and self-reported physical function six weeks after surgery. Furthermore, the authors highlighted the potential benefits of this approach in improving health-related quality of life and reducing psychological symptoms [29]. These findings underscore the significance of incorporating technology-based approaches that integrate the physical, emotional, and social dimensions of rehabilitation.

Conversely, another study detailing the development, content, and cost-effectiveness of an integrated rehabilitation care program within a personalized eHealth application for patients after knee arthroplasty found that eHealth interventions can enhance patients’ quality of life by reducing the time required to resume daily activities [30].

Patient satisfaction with rehabilitation nursing care, as measured by the QSEnf-10 scale, was high (3.8/4), indicating that the program effectively meets users’ needs. Key factors contributing to this satisfaction include continuous support, prompt responsiveness to patient needs, easy access to relevant information, and effective communication with the healthcare team. These elements not only reduce anxiety but also increase patients’ confidence in the recovery process. In this context, the “two-way communication” feature integrated into the +PERTO^®^ program, which allows patients to directly pose questions to the healthcare team, has proven to be particularly important for recovery. This assertion is supported by existing research demonstrating that direct communication channels between patients and healthcare professionals enhance both patient experience and clinical outcomes [12,31]. Thus, digital interventions not only improve clinical outcomes but also enrich the patient’s experience by facilitating more personalized and intimate monitoring throughout the recovery process.

Another important aspect is the observation that a significant proportion of patients support the integration of in-person and digital rehabilitation modalities, thereby reinforcing the comprehensive approach of the +PERTO^®^ program in postoperative rehabilitation. An international study aimed at evaluating patients’ perceptions of remote technologies for rehabilitation after total knee arthroplasty found that remote monitoring technologies were easy to use and motivating during the rehabilitation process [32], which are findings that were also reflected in this study.

The findings demonstrated that the +PERTO^®^ program exhibited both high usability and learnability, as evidenced by average scores of 96.3 and 97.7, respectively. These results signify an exemplary level of performance. The overall mean SUS score of 96.6 indicates that patients found the platform straightforward to utilize and that it was sufficiently adapted to their rehabilitation requirements. This is of particular significance in a context where treatment adherence is paramount to the efficacy of rehabilitation.

The high score in the learning ability domain indicates that patients can effectively use the program, which is essential for rehabilitation interventions that require continuity. This finding is consistent with previous research highlighting the importance of ease of use, learnability, and memorability in mobile health applications to promote patient adherence to treatment and postoperative recommendations [33]. Notably, this high score suggests that most patients were able to quickly familiarize themselves with the +PERTO^®^ program’s app, thereby facilitating their integration into the rehabilitation process.

eHealth interventions, such as the +PERTO^®^ program, enhance self-care, treatment adherence, and recovery, thereby improving system efficiency and reducing healthcare costs; these are critical components of modern rehabilitation and healthcare resource management [30].

In another relevant study, researchers assessed the impact of treatment continuity through a digital platform, finding that care continuity supported by digital technologies significantly enhances both functional recovery and emotional well-being in patients following total knee arthroplasty [34]. However, maintaining long-term patient adherence remains a recognized challenge in digital interventions [35]. To address this issue, the +PERTO^®^ program has been designed with continuous empowerment strategies and personalized rehabilitation programs, ensuring that patients receive adequate support throughout all stages of their recovery.

The use of technologies like the +PERTO^®^ program, along with other applications, can significantly enhance patient experience and satisfaction during the perioperative period [33]. The inclusion of videos, explanatory images, and reminders in the +PERTO^®^ program actively engages patients in their rehabilitation process [29].

Another important issue is the disparity in access to preoperative education, especially for patients living in rural or remote fields [36]. In this context, the +PERTO^®^ program deserves recognition for its commitment to providing equitable access to rehabilitation care, regardless of geographical location. This feature has the potential to reduce these disparities, ensuring that all patients have equal opportunities for preparation, knowledge acquisition, and recovery.

The findings suggest that the +PERTO^®^ program possesses the capacity to enhance the patient experience and minimize unscheduled hospital visits, thereby corroborating the evidence that mobile applications can play a pivotal role in the effective management of postoperative recovery. By offering continuous and personalized support, promoting self-care and adherence to rehabilitation protocols [33], and reducing costs [37], the +PERTO^®^ program demonstrates its potential to improve patient outcomes and reduce healthcare expenditure.

### 4.1. Limitations

Despite the promising results, this study has several limitations. The small sample size (*n* = 11) makes it difficult to generalize the findings to the broader population of total knee arthroplasty patients. A larger, more diverse sample would better reflect patient experiences and reduce statistical bias, making it easier to detect subtle differences or patterns.

Selection bias is another limitation. Participants may have been more motivated, more comfortable with digital technology, or from higher socioeconomic backgrounds, which may not reflect the general population. This could have influenced adherence to the program and affected the study’s external validity.

The six-week follow-up period is also a limitation, as it does not allow for assessing whether the benefits of the +PERTO^®^ program are sustained over time. Additionally, the lack of a control group prevents direct comparison with conventional rehabilitation methods. Without a comparison group, it is unclear whether the observed improvements are solely due to the +PERTO^®^ program or other factors, such as the placebo effect, natural recovery, or additional interventions. The absence of randomization further limits the ability to establish a clear cause-and-effect relationship.

Despite these limitations, this study serves as a pilot trial aimed at exploring the preliminary effects of the +PERTO^®^ program and its feasibility in total knee arthroplasty patients. The findings suggest a positive trend, indicating that the +PERTO^®^ program may offer meaningful clinical benefits.

### 4.2. Recommendations for Future Studies

To confirm and expand the results of this study, future research should include larger and more diverse samples. Multicenter studies involving patients with different demographic and clinical characteristics are recommended to enhance the generalizability of the findings. Conducting randomized controlled trials is essential to compare the effectiveness of the +PERTO^®^ program with traditional rehabilitation methods, allowing for more robust causal relationships to be established.

Long-term studies are also needed to determine whether the benefits of the +PERTO^®^ program last over time, particularly in terms of knee function and overall quality of life. Tracking patient adherence and identifying factors that influence the program’s success or challenges will help improve its effectiveness.

Since this study did not find significant improvements in social functioning, emotional well-being, or mental health, future interventions could include psychological support, emotional management strategies, and programs that encourage social interaction. These additions could promote a more well-rounded, patient-centered recovery.

Finally, economic analyses should be conducted to assess the cost-effectiveness of the +PERTO^®^ program. Research on its financial impacts, such as potential reductions in hospital readmissions, complications, and overall healthcare costs, could help policymakers justify investing in digital rehabilitation technologies.

## 5. Conclusions

This pilot study assessed the effects and usability of the +PERTO^®^ program, a Technological Rehabilitation Nursing Program, in patients undergoing total knee arthroplasty, demonstrating that a digital and accessible approach can be an effective solution for the follow-up and rehabilitation of these patients.

The implementation of the program highlighted its significant impact on pain reduction, overall knee function, and improved quality of life, with concurrent benefits in user experience and satisfaction. Additionally, high usability levels were confirmed.

As this is a pilot study, whose results justify the continued implementation of the +PERTO^®^ program; it is necessary to conduct randomized clinical trials as well as cost-effectiveness studies.

Despite the limitations of this initial study, the findings underscore the potential of digital interventions to optimize postoperative outcomes. The continuous and personalized use of the +PERTO^®^ program may provide a viable solution for minimizing complications, readmissions, and hospital costs by offering additional support beyond conventional practices. Integrating the +PERTO^®^ program into clinical practice represents a significant advancement in postoperative rehabilitation, fostering a more efficient, patient-centered recovery that aligns with the digitalization trends in healthcare. Overall, the findings emphasize the importance of incorporating digital technologies into healthcare to enhance accessibility, quality, and effectiveness, which are crucial for ensuring the sustainability of healthcare services.

## Figures and Tables

**Table 1:** Phases of the +PERTO Program.

Phase	Period	Main Activities
Phase 1	The healthcare team expects the postoperative phase to last two to four weeks.	Before undergoing surgery, patients must attend a preoperative consultation. During this consultation, the healthcare team trains patients on how to use the app, which includes instructions for starting daily exercises. The app also provides patients with access to useful information and facilitates communication with the healthcare team.
Phase 2	Hospitalization	The rehabilitation team bases the in-person rehabilitation protocol on disseminating pertinent information through mobile applications.
Phase 3	From hospital discharge to six weeks post-surgery.	The protocol includes continuing daily exercises at home, sending motivational messages, using decision-making algorithms, and facilitating communication with the healthcare team.

**Table 2:** Results obtained in relation to the OKS, SPPB, and SF36v2.

Instrument	Domain	T0 Median (IQR)	T1 Median (IQR)	Z Value	*p* Value *	*r* Value *
OKS	Pain	39.00 (25.00–57.00)	68.00 (61.00–75.00)	−2.807	0.005	0.846
	Function	50.00 (35.00–65.00)	65.00 (50.00–70.00)	−1.387	0.165	0.418
	Total OKS Score	23.00 (13.00–28.00)	30.00 (29.00–35.00)	−2.585	0.010	0.778
SPPB	Total Balance Test	4.00 (4.00–2.00)	4.00 (4.00–4.00)	−2.070	0.038	0.624
	Chair Stand Test	1.00 (3.00–1.00)	2.00 (4.00–1.00)	−0.844	0.399	0.254
	Gait Speed Test	2.00 (3.00–1.00)	2.00 (4.00–1.00)	−0.061	0.951	0.018
SF36v2	Physical Functioning	35.00 (55.00–25.00)	65.00 (80.00–60.00)	−2.937	0.003	0.885
	Physical Role	25.00 (44.00–25.00)	50.00 (63.00–38.00)	−2.251	0.024	0.678
	Bodily Pain	31.00 (41.00–22.00)	62.00 (72.00–41.00)	−2.547	0.011	0.768
	General Health	45.00 (60.00–25.00)	77.00 (87.00–60.00)	−2.936	0.003	0.885
	Vitality	44.00 (44.00–25.00)	63.00 (75.00–50.00)	−2.552	0.011	0.769
	Social Functioning	63.00 (100.00–38.00)	75.00 (100.00–50.00)	−1.382	0.167	0.416
	Limitations Due to Emotional Problems	33.00 (67.00–25.00)	50.00 (67.00–42.00)	−0.922	0.357	0.278
	Mental Health	55.00 (70.00–35.00)	70.00 (90.00–50.00)	−1.480	0.139	0.446
	Change in Health Status	2.00 (3.00–1.00)	4.00 (5.00–3.00)	−2.379	0.017	0.717

IQR—Interquartile Range; OKS—Oxford Knee Score; SPPB—Short Physical Performance Battery; SF36v2—MOS Short Form Health Survey 36 Item v2; * Wilcoxon test.

**Table 3:** Results related to satisfaction with rehabilitation nursing care.

Instrument	Item	Mean	SD	Median	Minimum	Maximum	IQR
QSEnf-10	1. Interpersonal relations towards you (politeness, respect, kindness, patience, care)	3.91	0.302	4.00	3	4	(4.00–4.00)
	2. Interest paid to you as a person and not only to your illness	3.82	0.405	4.00	3	4	(4.00–4.00)
	3. Capability to reassure you (comfort and support you received)	3.91	0.302	4.00	3	4	(4.00–4.00)
	4. Amount of time dedicated to you	3.82	0.405	4.00	3	4	(4.00–4.00)
	5. Speed of response to your calls	3.82	0.405	4.00	3	4	(4.00–4.00)
	6. Clarity of the information you received	3.82	0.405	4.00	3	4	(4.00–4.00)
	7. Amount of information you received	3.82	0.405	4.00	3	4	(4.00–4.00)
	8. Professionalism shown (skill, precision, etc.)	3.82	0.405	4.00	3	4	(4.00–4.00)
	9. Co-organization of the nursing team’s work	3.82	0.405	4.00	3	4	(4.00–4.00)
	10. Agreement among the nurses (harmony, collaboration, good mood)	3.82	0.405	4.00	3	4	(4.00–4.00)
Total		3.8	0.4	-	-	-	-

QSEnf-10—Nursing Care Satisfaction Questionnaire-10; SD—Standard Deviation; IQR—Interquartile Range.

**Table 4:** Individual distribution of the participants’ responses to the System Usability Scale (*n* = 11).

Participants	Q1 ^†^	Q2 ^†^	Q3 ^†^	Q4 ^†^	Q5 ^†^	Q6 ^†^	Q7 ^†^	Q8 ^†^	Q9 ^†^	Q10 ^†^	SUS Score ^±^
P1 ^§^	4	4	4	3	4	4	3	3	4	3	90
P2 ^§^	4	4	4	4	4	4	3	4	4	4	97.5
P3 ^§^	4	4	4	4	4	4	4	4	4	4	100
P4 ^§^	4	4	4	4	4	4	4	4	4	4	100
P5 ^§^	4	4	3	4	4	4	3	4	4	4	95
P6 ^§^	4	4	4	4	4	4	4	4	4	4	100
P7 ^§^	4	4	4	4	4	4	2	4	4	4	95
P8 ^§^	4	4	3	4	4	4	3	4	4	4	95
P9 ^§^	4	4	4	4	4	4	1	4	4	4	92.5
P10 ^§^	4	4	3	4	4	4	4	4	4	4	97.5
P11 ^§^	4	4	4	4	4	4	4	4	4	4	100
Overall, SUS average ^±^											**96.6**

^†^ Q = System Usability Scale question; ^§^ P = participant; ^±^ SUS Score = questionnaire’s System Usability Scale score.

**Table 5:** SUS subscales/dimensions.

Dimensions	Questions	Minimum	Maximum	Mean	SD
Usability	Q1; Q2; Q3; Q5; Q6; Q7; Q8; Q9	90.6	100	96.3	3.37
Learning ability	Q4; Q10	75	100	97.7	7.5

SUS—System Usability Scale; SD—Standard Deviation.

## Data Availability

The data used in this study are available upon request. To access the dataset, interested parties must justify the need for its use and contact the corresponding author. Access will be subject to ethical and regulatory considerations and will be granted only for legitimate research purposes.
